# Interventional bronchoscopy in malignant central airway obstruction by extra-pulmonary malignancy

**DOI:** 10.1186/s12890-018-0608-6

**Published:** 2018-03-13

**Authors:** Beomsu Shin, Boksoon Chang, Hojoong Kim, Byeong-Ho Jeong

**Affiliations:** 1 Department of Internal Medicine, Yonsei University Wonju College of Medicine, Wonju, South Korea; 2 Department of Pulmonary and Critical Care Medicine, Kyung Hee University Hospital at Gangdong, School of Medicine, Kyung Hee University, Seoul, South Korea; 3 Division of Pulmonary and Critical Care Medicine, Department of Internal Medicine, Samsung Medical Center, Sungkyunkwan University School of Medicine, 81 Irwon-ro, Gangnam-gu, Seoul, 06351 South Korea

**Keywords:** Interventional bronchoscopy, Malignant central airway obstruction, Extra-pulmonary malignancy

## Abstract

**Background:**

Interventional bronchoscopy is considered an effective treatment option for malignant central airway obstruction (MCAO). However, there are few reports of interventional bronchoscopy in patients with MCAOs due to extra-pulmonary malignancy. Therefore, the objective of this study was to investigate treatment outcomes and prognostic factors for bronchoscopic intervention in patients with MCAO due to extra-pulmonary malignancy.

**Methods:**

We retrospectively analyzed consecutive 98 patients with MCAO due to extra-pulmonary malignancy who underwent interventional bronchoscopy between 2004 and 2014 at Samsung Medical Center (Seoul, Korea).

**Results:**

The most common primary site of malignancy was esophageal cancer (37.9%), followed by thyroid cancer (16.3%) and head & neck cancer (10.2%). Bronchoscopic interventions were usually performed using a combination of mechanical debulking (84.7%), stent insertion (70.4%), and laser cauterization (37.8%). Of 98 patients, 76 (77.6%) patients had MCAO due to progression of malignancy, and 42 (42.9%) patients had exhausted all other anti-cancer treatment at the time of bronchoscopic intervention. Technical success was achieved in 89.9% of patients, and acute complications and procedure-related deaths occurred in 20.4% and 3.1% of patients, respectively. Reduced survival was associated with MCAO due to cancer other than thyroid cancer or lymphoma, mixed lesions, and not receiving adjuvant treatment after bronchoscopic intervention.

**Conclusions:**

Bronchoscopic intervention could be a safe and effective procedure for MCAO due to end-stage extra-pulmonary malignancies. In addition, we identified possible prognostic factors for poor survival after intervention, which could guide clinicians select candidates that will benefit from bronchoscopic intervention.

## Background

Malignant central airway obstruction (MCAO) is clinically significant and is usually considered as a criterion for intervention; it is usually defined as ≥50% occlusion of the cross-sectional area of the trachea, mainstem bronchi, bronchus intermedius, or lobar bronchus [1]. MCAO can cause dyspnea, hemoptysis, atelectasis, and obstructive pneumonia, and is associated with decreased quality of life and increased mortality [2, 3]. However, treatments to resolve MCAOs have many limitations. Surgery is restrictively performed according to the location of the lesion, stage of the disease, and the general medical condition of each patient [4]. In addition, long periods of chemotherapy and radiation therapy may be needed to resolve airway obstruction [5]. On the other hand, bronchoscopic intervention can immediately alleviate symptoms related to airway obstruction and improve quality of life [1, 6, 7].

In total, 20–50% of patients with extra-pulmonary malignancy will have lung metastasis during their clinical course [8]. In addition, endobronchial metastases may occur in 1–4% of patients with extra-pulmonary malignancy [9, 10]. Finally, extra-pulmonary malignancy comprises 8–40% of all MCAO cases [1, 11, 12]. However, most studies have conducted analyses without classification based on primary malignancy sites, such as pulmonary and extra-pulmonary malignancies [1, 6, 11–15]. Based on these reports, there are limitations when managing patients with MCAO from an extra-pulmonary malignancy.

Herein, we conducted a retrospective study to evaluate the clinical outcomes and prognostic factors of bronchoscopic interventions in patients with MCAO due to extra-pulmonary malignancy.

## Methods

### Patients

We retrospectively reviewed all interventional bronchoscopy reports from January 2004 to December 2014 at Samsung Medical Center (a 1979-bed, tertiary care referral hospital in Seoul, South Korea) and identified 98 patients with MCAO due to extra-pulmonary malignancy. This study obtained approval from the institutional review board (IRB no. 2017-01-033) to review and publish information obtained from patient records. The need for informed consent was waived because patient information was anonymized and de-identified prior to analysis.

### Airway intervention techniques

Airway anatomy was evaluated using chest computed tomography (CT) and, when possible, flexible bronchoscopy. MCAO was defined as ≥50% occlusion of the cross-sectional area of the trachea, main stem bronchi, bronchus intermedius, or lobar bronchus on the CT images or bronchoscopic findings [1].

Interventional bronchoscopy was performed according to standard techniques [16, 17]. After induction of general anesthesia, the patient was intubated with a rigid bronchoscope tube (Bryan Co., Woburn, MA, USA or Karl-Storz, Tuttlingen, Germany). Depending on the characteristics of airway obstruction (endobronchial lesion, extrinsic compression, and mixed lesion) and the medical condition of the patient, various combinations of airway intervention techniques were used, including mechanical debulking, laser, and insertion of silicone stents. Any endobronchial lesion was removed mechanically using rigid bronchoscope tubes and forceps. A neodymium-doped yttrium aluminum garnet laser (LaserSonics, Milpitas, CA, USA) or a diode laser (Biolitec, Ceralas^®^, Germany) were used to cauterize visible vessels before mechanical debulking or to ablate residual endobronchial tumor. Additionally, if needed, a silicone stent (Natural stent [M1S Co., Seoul, Korea] or Dumon stent [Novatech, La Ciotat, France]) was inserted to maintain airway patency against extrinsic compression or rapid progression [18].

Technical success was based on anatomic criteria, which were defined as the reopening of the airway lumen to > 50% of the normal cross sectional area and a connection to a viable area of distal lung by bronchoscopy [1]. If a physician successfully reopened a proximal airway, but then discovered distal lesions that occluded all segmental or subsegmental levels, this was classified as a technical failure [1].

### Data collection

We retrospectively reviewed the medical records of all 98 patients. The following data were collected: demographic characteristics, primary site of malignancy, bronchoscopic findings (site of lesion, type of obstruction, and severity and length of stenosis), pre- and post-procedure treatment modalities, techniques applied during bronchoscopic interventions, procedure-related complications, and survival time.

Performance status was evaluated using the American Society of Anesthesiologists (ASA) physical status classification. Poor performance was defined as ≥ class 3 ASA physical status, which indicates severe systemic disease with functional limitation [19]. The severity of airway stenosis was determined using the Myer-Cotton stenosis grading system: Grade I, ≤50% luminal stenosis; Grade II, 51–70% luminal stenosis; Grade III, 71–99% luminal stenosis; and Grade IV, no lumen [20]. Respiratory distress was defined as worsening oxygenation or dyspnea after the procedure requiring additional oxygen supplies for at least 24 h. Excessive bleeding was defined as procedure-related bleeding which required transfusion or escalation in level of care.

Because all patients were in terminal stage with pulmonary metastasis from extra-pulmonary malignancy, the status of malignancy at the first bronchoscopic intervention was divided as follows. First, detection time of MCAO was divided as initial diagnosis of malignancy that had not yet been treated and disease progression of malignancy despite anti-cancer treatment. Second, we investigated the history of anti-cancer treatment after the first bronchoscopic intervention, and patients were divided into whether they received adjuvant treatment or not.

### Statistical analysis

Data are presented as number (%) for categorical variables and median (interquartile range [IQR]) for continuous variables. The Kaplan–Meier method was used to estimate overall survival after the first bronchoscopic intervention. A multivariable Cox proportional hazard regression analysis was used to identify independent prognostic factors associated with overall survival. Because the total number of patients was not large enough to allow all variables to be analyzed, demographic data and variables that seemed to be directly related to the procedure and survival were selectively included in the analysis. The appropriateness of the proportional hazards assumption was evaluated using plots of the log minus log of Kaplan–Meier survival against the log of time. Statistical differences were considered significant at *P* < 0.05. All statistical analyses were performed using SPSS software (IBM SPSS Statistics ver. 22, Chicago, IL, USA).

## Results

### Baseline characteristics

Baseline characteristics of the study population are summarized in Table 1. Of the 98 patients, 60 (61.2%) were males. The median age was 63 (IQR, 54–72) years old and the median body mass index (BMI) was 21.1 kg/m^2^ (IQR, 18.1–24.3 kg/m^2^). Thirty-five patients (35.7%) had an ASA physical status ≥ class 3, and intubation before intervention was needed in 12 (12.2%) patients due to respiratory failure. The most common primary site of malignancy was esophageal cancer (37.9%), followed by thyroid cancer (16.3%), head & neck cancer (10.2%), renal cell carcinoma (7.1%), colorectal cancer (7.1%), and lymphoma (5.1%).

**Table 1:** Baseline characteristics

Variables	*N* = 98
Age, years	63 (54–72)
Sex, male	60 (61.2)
Body mass index, kg/m^2^	21.1 (18.1–24.3)
Comorbidity	
Diabetes mellitus	10 (10.2)
Chronic liver disease	8 (8.2)
Cerebrovascular disease	7 (7.1)
Congestive heart disease	6 (6.1)
Chronic pulmonary disease	3 (3.1)
Poor performance status^a^	35 (35.7)
Intubation due to respiratory failure before intervention	12 (12.2)
Primary site of malignancy	
Local extension	63 (64.4)
Esophageal cancer	37 (37.9)
Thyroid cancer	16 (16.3)
Head & neck cancer	10 (10.2)
Metastatic disease	35 (35.6)
Renal cell carcinoma	7 (7.1)
Colorectal cancer	7 (7.1)
Lymphoma	5 (5.1)
Others^b^	16 (16.3)

Data are presented as n (%) or the median (interquartile range)

^a^American Society of Anesthesiologists (ASA) physical status class ≥3 means severe systemic disease with functional limitation

^b^Sarcoma (*n* = 4), breast cancer (*n* = 3), hepatocellular carcinoma (*n* = 3), gastric cancer (*n* = 1), ovarian cancer (*n* = 1), neuroblastoma (*n* = 1), thymic cancer (*n* = 1), mesothelioma (*n* = 1), and peripheral nerve sheath tumor (*n* = 1)

Characteristics of the MCAO site are summarized in Table 2. The most common site of MCAO was the trachea (63.3%), followed by the left main bronchus (21.4%) and the right main bronchus (6.1%). Mixed, endobronchial, and extrinsic obstructions were seen in 54 (55.1%), 29 (29.6%), and 15 (15.3%) patients, respectively. Most patients (74.5%) had ≥71% obstruction of cross sectional area (Grade III or IV). The median length of stenosis was 30 mm (IQR, 22–38 mm). Some patients (8.2%) had a fistula between the trachea and the esophagus.

**Table 2:** Bronchoscopic findings

Variables	*N* = 98
Site of lesion	
Single lesion	93 (94.9)
Trachea	62 (63.3)
Left main bronchus	21 (21.4)
Right main bronchus	6 (6.1)
Right bronchus intermedius	1 (1.0)
Lobar bronchus	3 (3.1)
Extended lesion	5 (5.1)
Trachea and each or both bronchi	3 (3.1)
Both main bronchi	2 (2.0)
Type of obstruction	
Endobronchial lesion	29 (29.6)
Extrinsic compression	15 (15.3)
Mixed lesion	54 (55.1)
Severity of stenosis (Myer and Cotton Grade)^a^	
II	25 (25.5)
III	55 (56.1)
IV	18 (18.4)
Length of MCAO^b^, mm	30 (22–38)
Fistula between trachea and esophagus	8 (8.2)

Data are presented as n (%) or the median (interquartile range)

*MCAO* malignant central airway obstruction

^a^Categorization based on the percentage of reduction in cross-sectional area. Grade 1, ≤ 50% lumenal stenosis; Grade II, 51–70% lumenal stenosis; Grade III, 71–99% lumenal stenosis; Grade IV, no lumen

^b^Length of MCAO was defined as the sum of the length of the obstructive lesions more than Grade II

### Treatment modalities and complications

The median time from diagnosis of MCAO to bronchoscopic intervention was 0.4 months (IQR, 0.2-1.0 months) (Table 3). Bronchoscopic interventions were usually performed using a combination of treatment modalities, including mechanical debulking (84.7%), stent insertion (70.4%), and laser cauterization (37.8%). Thirty-five (35.7%) patients underwent bronchoscopic intervention more than twice during their clinical courses. Finally, only 10 patients (10.2%) had a technical failure. Of 22 patients who were diagnosed with MCAO as the initial diagnosis of malignancy, 7 (31.8%) patients did not receive adjuvant treatment after interventional bronchoscopy. Of 76 patients who were diagnosed with MCAO due to progression of their malignancy, 35 (46.1%) did not receive adjuvant treatment after interventional bronchoscopy.

**Table 3:** Treatment modalities and complications

Variables	*N* = 98
Time interval from diagnosis of MCAO to intervention, months	0.4 (0.2 – 1.0)
Treatment modalities	
Mechanical debulking	83 (84.7)
Silicone stent	69 (70.4)
Tube stent	65 (66.3)
Y stent	7 (7.1)
Laser	37 (37.8)
Tracheostomy	4 (4.1)
Number of interventional bronchoscopies	
1	63 (64.3)
≥ 2	35 (35.7)
Technical failure	10 (10.2)
MCAO as initial diagnosis of malignancy	22 (22.4)
No adjuvant treatment after interventional bronchoscopy	7 (7.1)
Adjuvant radiation therapy	10 (10.2)
Adjuvant chemotherapy	6 (6.1)
Adjuvant surgical resection	2 (2.0)
MCAO as disease progression of malignancy	76 (77.6)
No adjuvant treatment after interventional bronchoscopy	35 (35.7)
Adjuvant radiation therapy	28 (28.6)
Adjuvant chemotherapy	18 (18.4)
Adjuvant surgical resection	1 (1.0)
Acute complications	20 (20.4)
Respiratory distress	13 (13.3)
Excessive bleeding	10 (10.2)
Pneumothorax	3 (3.1)
Procedure-related death^a^	3 (3.1)
30-day mortality	13 (13.3)
Chronic complications	25 (25.5)
Mucostasis	19 (19.4)
Granulation tissue overgrowth	10 (10.2)
Stent migration	6 (6.1)

Data are presented as n (%) or the median (interquartile range)

*MCAO* malignant central airway obstruction

Patients could undergo more than one adjuvant treatment

Patients could have more than one complication

^a^Three patients died from tension pneumothorax, excessive bleeding, and pneumonia, respectively

Acute complications included respiratory distress (*n* = 13), excessive bleeding (*n* = 10), and pneumothorax (*n* = 3) (Table 3). Procedure-related deaths occurred in three patients from tension pneumothorax, excessive bleeding, and pneumonia, respectively. Although chronic complications including mucostasis (*n* = 19), granulation tissue overgrowth (*n* = 10), and stent migration (*n* = 6) occurred not infrequently, most complications were manageable with additional procedures.

### Survival and prognosis

**Fig. 1:**
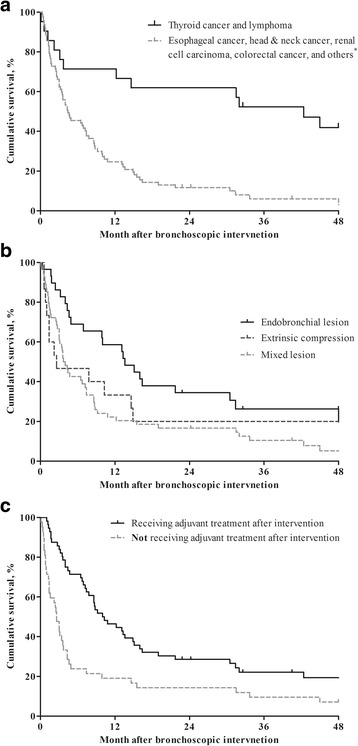
Overall survival of patients after interventional bronchoscopy. Survival based on (**a**) primary site of malignancy, (**b**) type of obstruction, and (**c**) receiving adjuvant treatments or not after interventional bronchoscopy. MCAO, malignant central airway obstruction. ^*^Sarcoma (*n* = 4), breast cancer (*n* = 3), hepatocellular carcinoma (*n* = 3), gastric cancer (*n* = 1), ovarian cancer (*n* = 1), neuroblastoma (*n* = 1), thymic cancer (*n* = 1), mesothelioma (*n* = 1), and peripheral nerve sheath tumor (*n* = 1)

Figure 1 shows the overall survival after bronchoscopic intervention according to primary site of malignancy, type of obstruction, and adjuvant treatment or not after interventional bronchoscopy. Median survival was 7 months and 30-day mortality was 13.3%. Survival rates at 1 year, 2 years, and 5 years were 34.7%, 22.4%, and 11.2%, respectively.

Table 4 shows independent prognostic factors related to mortality based on analysis with univariate and multivariate Cox proportional hazard regression. Because the plots of the log minus log of Kaplan–Meier survival against the log of time for all the variables seemed to be parallel, the hazards can be considered proportional. According to these analyses, overall survival was independently associated with primary site of malignancy, type of obstruction, and adjuvant treatment. Patients with thyroid cancer or lymphoma had better survival than those with other primary malignancies (adjusted hazard ratio [aHR], 0.245; 95% confidence interval [CI], 0.104–0.573; *P* = 0.001). Mixed lesions were significantly associated with worse survival compared to endobronchial lesions (aHR, 1.951; 95% CI, 1.084–3.510; *P* = 0.026). Patients with MCAO receiving adjuvant treatment had better survival than those with MCAO not receiving adjuvant treatment (aHR, 0.519; 95% CI, 0.301–0.895; *P* = 0.018).

**Table 4:** Prognostic factors related to mortality

Variables	*N*	Univariable Cox regression		Multivariable Cox regression	
		Unadjusted HR (95% CI)	*P*	Adjusted HR (95% CI)	*P*
Age, years	–	0.996 (0.982–1.010)	0.596	1.005 (0.981 – 1.029)	0.679
Sex, male	60	1.224 (0.792–1.890)	0.363	0.721 (0.398 – 1.307)	0.282
Body mass index, kg/m^2^	–	0.925 (0.869–0.984)	0.013	0.968 (0.901 – 1.041)	0.381
Poor performance status^a^	35	1.201 (0.776–1.859)	0.411	0.801 (0.470 – 1.363)	0.413
Intubation due to respiratory failure before intervention	12	1.308 (0.692–2.470)	0.409	1.110 (0.517 – 2.384)	0.788
Primary site of malignancy					
Esophageal, head & neck, renal, and colorectal cancer and others^b^	77	Reference		Reference	
Thyroid cancer and lymphoma	21	0.316 (0.174–0.575)	< 0.001	0.245 (0.104–0.573)	0.001
Site of lesion					
Single lesion	93	Reference		Reference	
Extended lesion	5	3.399 (1.333–8.663)	0.010	1.407 (0.433 – 4.571)	0.571
Type of obstruction					
Endobronchial lesion	29	Reference		Reference	
Extrinsic compression	15	1.261 (0.624–2.551)	0.518	0.879 (0.321–2.404)	0.802
Mixed lesion	54	1.914 (1.171–3.130)	0.010	1.951 (1.084–3.510)	0.026
Severity of stenosis (Myer and Cotton Grade)^c^					
II and III	80	Reference		Reference	
IV	18	1.057 (0.617–1.809)	0.841	0.556 (0.276 – 1.120)	0.100
Length of MCAO^d^, mm	–	1.007 (0.989–1.024)	0.468	1.016 (0.988 – 1.044)	0.274
Number of interventional bronchoscopies					
1	63	Reference		Reference	
≥ 2	35	0.721 (0.461–1.129)	0.153	0.787 (0.461 – 1.342)	0.378
Detection time of MCAO					
Initial diagnosis of malignancy that have not yet been treated	22	Reference		Reference	
Disease progression of malignancy despite anti-cancer treatment	76	1.963 (1.146–3.362)	0.014	1.541 (0.710 – 3.347)	0.274
Adjuvant treatment after interventional bronchoscopy					
Not receiving adjuvant treatment	56	Reference		Reference	
Receiving adjuvant treatment	42	0.501 (0.327–0.768)	0.002	0.519 (0.301–0.895)	0.018

*HR* hazard ratio, *CI* confidential interval, *MCAO* malignant central airway obstruction

^a^American Society of Anesthesiologists (ASA) physical status class ≥3 means severe systemic disease with functional limitation

^b^Sarcoma (*n* = 4), breast cancer (*n* = 3), hepatocellular carcinoma (*n* = 3), gastric cancer (*n* = 1), ovarian cancer (*n* = 1), neuroblastoma (*n* = 1), thymic cancer (*n* = 1), mesothelioma (*n* = 1), and peripheral nerve sheath tumor (*n* = 1)

^c^Categorization based on the percentage of reduction in cross-sectional area. Grade 1, ≤ 50% lumenal stenosis; Grade II, 51–70% lumenal stenosis; Grade III, 71–99% lumenal stenosis; Grade IV, no lumen

^d^Length of MCAO was defined as the sum of the length of the obstructive lesions more than Grade II

## Discussion

In patients with MCAO, bronchoscopic intervention can provide significant palliation [1, 21, 22]. In particular, bronchoscopic intervention may relieve life-threatening obstruction and provide better opportunities for other therapeutic modalities such as radiation and chemotherapy in patients with respiratory failure caused by MCAO [23]. This study revealed that bronchoscopic interventions such as mechanical debulking, laser, and stent insertion could be performed safely and successfully in most patients with MCAO due to extra-pulmonary malignancy. In addition, we found that poor survival was associated with primary malignancy site, mixed lesions, and not receiving adjuvant treatment after interventional bronchoscopy.

Recently, cancer survival has consistently increased because of advances in early detection and treatment and aging of the population [24]. In this respect, MCAO is importantly associated with a major reduction in quality of life and survival [3]. As the effectiveness of bronchoscopic intervention in patients with MCAO is well-known [1, 6, 11–14, 25, 26], bronchoscopic intervention is a preferred palliative therapy for relief of MCAO [21, 22]. However, most research is limited to patients with MCAO due to primary pulmonary malignancy [25, 26], or to a small proportion of patients with MCAO due to extra-pulmonary malignancy [1, 6, 11–15]. Furthermore, previous studies performed analysis without separating patients based on the primary site of malignancy. Although some reports targeted patients with MCAO due to extra-pulmonary malignancy, only a small number of patients were included [27–29]. For these reasons, previous reports were limited when analyzing treatment outcomes and prognostic factors in patients with MCAO due to extra-pulmonary malignancy.

In the present study, the technical success rate was 90.8%, and acute complication- and procedure-related mortality were 20.4% and 3.1%, respectively. Because there are few reports regarding bronchoscopic intervention in patients with MCAO due to extra-pulmonary malignancy, it is difficult to compare clinical outcomes with previous reports. Nonetheless, the technical success rate of the present study was as high as previous reports of interventional bronchoscopy in patients with MCAO due to mainly primary pulmonary malignancy, which was 88–100% [1, 6, 14, 25, 26]. However, acute complications and procedure-related mortality were slightly higher in the present study than in previous studies, which were 3–10% [6, 11, 25, 30] and 1% [6, 12, 14, 30], respectively. Considering that most of our patients had terminal-stage cancer and almost half of the patients had no further options for anti-cancer treatment in this study, we think that these technical success rates and complication rates are reasonable.

In this study, we also investigated prognostic factors associated with overall survival after the first bronchoscopic intervention. First, survival depends on the primary site of malignancy. We expected that patients with MCAO due to thyroid cancer (which is known to be slowly progressive) and lymphoma (known to have good response to chemoradiation therapy) would have better survival than those with MCAO due to other malignancies. Second, mixed lesions were a poor prognostic factor compared to endobronchial lesions. Mixed lesions frequently require a multimodal approach and can be associated with increased complications and mortality [15, 30]. Third, patients with MCAO not receiving adjuvant treatment after interventional bronchoscopy had a poor prognosis. As seen in previous studies, survival was poor among patients who had exhausted all options such as radiation, chemotherapy, and surgery before bronchoscopic intervention [6, 15, 25]. On the other hand, BMI, poor performance status, as evaluated by ASA score, intubation state before intervention, and detection time of MCAO were not significantly associated with increased mortality in our study. We hypothesize that this was because their poor general condition was rapidly resolved and did not influence long term survival after successful intervention. Thus, bronchoscopic intervention should not be limited to these populations.

This report has several limitations. First, selection bias may have influenced the significance of our results due to the retrospective design at a single center. Second, there have been advancements in treatment options for patients with terminal malignancy, such as chemoradiotherapy and supportive care throughout the study period. These advancements might have influenced recent patients, leading to slower disease progression and better survival than former patients. Third, there may be a lead-time bias in our results. Because all the patients were in terminal stage with pulmonary metastasis from extra-pulmonary malignancy at the time of diagnosis for MCAO, we divided all patients by detection time and receiving adjuvant treatment or not. Although we adjusted for these variables in the multivariable Cox proportional hazard regression analysis, lead-time bias may have influenced our results. Fourth, the number of patients was relatively small for multivariate analysis, including many prognostic variables. Therefore, interpretation of the main results will require caution. Finally, we could not evaluate spirometric data, quality of life, and symptom scores before and after treatment. This would be vital since the interventional procedures for MCAO are primarily palliative.

## Conclusions

In conclusion, bronchoscopic intervention could be a safe and effective procedure for terminal stage cancer patients with extra-pulmonary malignancies with lung metastasis. Poor prognosis may be related with MCAO due to cancer other than thyroid cancer or lymphoma, mixed lesions, and no adjuvant treatment after bronchoscopic intervention. We hope that this study can help clinicians select candidates that will benefit from bronchoscopic intervention.
